# Knowledge, Attitudes, and Practices Regarding Cervical Cancer Screening among Omani Women Attending Primary Healthcare Centers in Oman: A Cross-Sectional Survey

**DOI:** 10.31557/APJCP.2021.22.3.775

**Published:** 2021-03

**Authors:** Tagharid Al Yahyai, Marwa Al Raisi, Rahma Al Kindi

**Affiliations:** 1 *Oman Medical Specialty Board, Muscat, Sultanate of Oman. *; 2 *Department of Family Medicine and Public Health, Sultan Qaboos University, Muscat, Sultanate of Oman. *

**Keywords:** Cervical cancer, papanicolaou test, screening- knowledge, attitudes, practice, Oman

## Abstract

**Background:**

This study aimed to assess knowledge, attitudes, and practices regarding cervical cancer, cervical cancer screening, and Papanicolaou (Pap) smear testing among Omani women attending primary healthcare centers in Oman, and to establish a correlation with various sociodemographic characteristics.

**Methods:**

A multi-center cross-sectional survey was carried out from August 2019 to January 2020 and included 805 women attending 18 primary healthcare centers. A pre-tested questionnaire was utilized to assess the participants’ sociodemographic characteristics, cervical cancer risk factors, knowledge, attitudes, and practices related to cervical cancer, cervical cancer screening, and Pap smear testing.

**Results:**

All 805 women participated in the study (response rate: 100%). Overall, 67.5% and 50.9% had heard of cervical cancer and Pap smear testing, respectively; however, only 13.4% and 10.9% demonstrated high levels of knowledge concerning these topics. Knowledge was significantly associated with educational level, type of educational qualification (i.e. if their degree was related to healthcare), monthly income, and employment status (p ≤ 0.05 each). Only 15.7% of the participants had previously undergone Pap smear testing, although 42.7% were willing to undertake such screening in future. No associations were noted between Pap smear practice or willingness and sociodemographic characteristics, family history of cervical cancer or personal history of cervical cancer or related risk factors.

**Conclusions:**

Knowledge regarding cervical cancer and Pap smear testing was suboptimal among a cohort of Omani women attending primary healthcare centers in Oman. This may be a factor behind the increased number of cervical cancer cases in Oman; as such, a well-structured awareness and educational program is needed to address this issue.

## Introduction

Cervical cancer is a type of cancer that affects the female reproductive system, namely the cervix (Sherris et al., 2001). Affected patients are usually asymptomatic in the early stages and then, as the disease progresses, present with abnormal vaginal bleeding, vaginal discomfort, malodorous discharge, and dysuria (Boardman, 2019). Globally, cervical cancer is the third most common cancer in women, with the highest incidence occurring in those aged 40–49 years (Arbyn et al., 2020). Although common worldwide, the burden of cervical cancer is greater in developing regions, with significant disparities in mortality due to economic, regional, and racial disparities (Sherris et al., 2001; Yoo et al., 2017).

Multiple factors have been found to play a role in cervical carcinogenesis, including human papillomavirus (HPV), duration of infection, the host’s immune status, environmental factors, and lack of access to routine cytology screening (Tsikouras et al., 2016; American Cancer Society, 2020). Other gynecological risk factors include early age at first sexual intercourse or first full-term pregnancy and having multiple sexual partners (Tsikouras et al., 2016; American Cancer Society, 2020). Moreover, there is a five-fold increase in the risk of cervical cancer among patients with human immunodeficiency virus (HIV) infections (Tsikouras et al., 2016; Boardman, 2019). 

Overall, HPV infection is the most commonly reported causative organism of cervical cancer and has been implicated in 99% of cervical malignancies, primarily squamous cell carcinomas and adenocarcinomas (Bosch et al., 2002). Of the 14 oncogenic HPV genotypes, types 16 and 18 constitute ~70% of all cervical cancers around the world (World Health Organization, 2019). However, although highly prevalent among sexually active women, 90% of HPV infections resolve spontaneously without complications within a few years (Boardman, 2019). On average, only 5% of infections will result in cervical intraepithelial neoplasia (CIN) grades 2 or 3-a recognized precursor of cervical cancer-within three years of infection, while 20% and 40% of cases of CIN grade 3 progress to invasive cervical cancer within five and 30 years, respectively (Boardman, 2019). 

Various international guidelines for cervical cancer screening exist, of which the majority recommend that screening be initiated for all sexually active women from the age of 21–25 years and continue until the age of 65–70 years (American Cancer Society, 2016; American College of Obstetricians and Gynecologists, 2016; National Health Service, 2016; Curry et al., 2018;). Such screening can be performed by Papanicolaou (Pap) smear testing alone every three years, or a combination of Pap smear testing and HPV screening every five years (National Health Service, 2016; Curry et al., 2018). Further tests and procedures, such as colposcopies and biopsies, can also be used to confirm the diagnosis (Tsikouras et al., 2016). Generally speaking, Pap smear testing is one of the most practical and effective tools for the screening and early detection of cervical cancer, particularly as it is easy to perform, cost-effective, and readily available; however, the uptake of such screening is generally low, particularly in developing countries (Boardman, 2019). 

In Oman, cervical cancer is the third most common cancer among women aged 15–44 years (Catalan Institute of Oncology/International Agency for Research on Cancer Information Centre on HPV and Cancer, 2019). Combined with the absence of a well-structured national screening program, lack of knowledge regarding the availability of the test is believed to be the main reasons for the high incidence of cervical cancer in this region (Ortashi et al., 2013; Ali et al., 2017). Previous research by Nasar et al., (2016) indicates that the majority of Omani women attending a tertiary teaching institute had heard of cervical cancer; however, the women lacked specific knowledge regarding cervical cancer signs and symptoms, risk factors, and Pap smear testing. As such, the present study aimed to assess knowledge, attitudes, and practices regarding cervical cancer and Pap smear screening among Omani women attending primary healthcare centers across the country, as well as to establish a correlation with various sociodemographic factors.

## Materials and Methods

*Study design*

A multi-center cross-sectional survey was carried out from August 2019 to January 2020 at 18 primary healthcare centers across all 11 governorates of Oman. The number of health centers varies from one governorate to another depending on the population and number of wilayats (counties) in each region. For the purpose of this study, two health centers were randomly selected from each of the highly populated governorates as well as one health center from the less populated governorates. A total of 805 women aged ≥18 years attending the selected health centers for various reasons during the study period were recruited via a systematic random sampling strategy in which every second woman was selected. However, those with learning difficulties, those who did not speak Arabic or English, and those with no time to participate in the survey were excluded.

*Sample size*

The necessary sample size was calculated to be 800, based on an anticipated level of knowledge regarding cervical cancer and its screening (50%), with a 5% margin of error, 95% confidence level, 5% alpha error, and a design effect of 2.

*Data collection*

The participants underwent face-to-face interviews during which a pre-tested and well-structured Arabic-language questionnaire was used for data collection. The questionnaire had been previously used in a similar study performed in Oman (Nasar et al., 2016). The interviews were conducted by trained researchers assisted by qualified physicians and took approximately 15–20 minutes.

The questionnaire was divided into four main sections. The first section covered the participants’ sociodemographic characteristics, including their age, education level, whether their degree was related to healthcare, employment status, marital status, age at first marriage, number of marriages, number of children and daughters, their husbands’ education level, monthly income, and if their financial status affected their ability to undertake regular gynecological examinations. The second section assessed the presence of known risk factors for cervical cancer, such as smoking status, exercise habits, use of combined oral contraceptive methods, history of abortions, sexually transmitted diseases (STDs), HPV infection, cervical cancer, family history of cervical cancer, and whether they or their husbands had been previously diagnosed with low immunity, defined as the presence of one or more of the following factors: a diagnosis of HIV or acquired immunodeficiency syndrome (AIDS), or a history of immunosuppressant drugs or organ transplantation. 

The third part of the questionnaire assessed the participants’ knowledge and attitudes regarding cervical cancer, including whether they had ever heard of cervical cancer and, if so, their source of information, and whether they believed cervical cancer to be among the top leading causes of cancer-related death worldwide. In addition, this section explored knowledge of warning signs of cervical cancer, such as vaginal bleeding between periods, during or after intercourse, or after menopause, persistent lower back pain, persistent foul-smelling vaginal discharge, discomfort, or pain during intercourse, heavy or prolonged menstrual periods, persistent diarrhea, persistent pelvic pain, blood in the stool or urine, and unexplained weight loss. Finally, the participants were asked to rate their agreement with potential risk factors of cervical cancer, including HPV infection, smoking, low immunity (e.g. because of HIV or AIDS, or as a result of immunosuppressant drugs or an organ transplant), having a husband with low immunity, long-term use of oral contraceptives pills, early marriage (i.e. before the age of 16 years), having many children (i.e. three or more), a family history of cervical cancer, and not undertaking regular screening. 

The last part of the questionnaire evaluated the participants’ knowledge, attitudes, and practices related to cervical cancer screening and Pap smear testing. Participants were asked if they had ever heard of Pap smear screening and, if so, their source of information. In addition, they were asked to identify the part of the body tested during a Pap smear, determine the ideal frequency and candidate for testing (e.g. if Pap smear testing is performed only for married women or only before menopause), assess the importance of Pap smear testing, explain how abnormal Pap smear results are interpreted, and comment on the availability of Pap smear testing in Oman. Several items were also included to determine barriers to screening, including whether the gender of the physician would affect their decision to undertake screening. 

Actual screening practices were also assessed, with participants asked if they had previously undergone Pap smear testing and, if so, their reasons for doing so, the last time they had undertaken screening, at what age they had first undertaken screening, and the frequency with which this screening was performed. In turn, those who had never undertaken Pap smear testing were asked their reasons for not doing so, their willingness to undertake screening within the coming six months, and whether they would be keen to undergo screening regularly. Finally, all participants were asked questions regarding the procedure for follow-up testing after receiving abnormal Pap smear results. 

*Scoring*

All items in the latter two sections of the questionnaire were compiled and scored as follows. The third section comprised four items assessing knowledge of cervical cancer, including two questions on general knowledge, one on warning signs, and one on risk factors, with the latter two questions listing 11 specific warning signs and nine risk factors of cervical cancer. Each correct answer received a score of one, resulting in a maximum score of 22. Total scores of <7, 7–14, and >14 were considered to indicate poor, acceptable, and high levels of knowledge, respectively. 

The fourth section included eight items related to knowledge of cervical cancer screening and Pap smear testing, of which the first seven were general knowledge questions worth one point each. The eighth item contained five questions related to knowledge regarding follow-up tests after abnormal Pap smear results. Each correct answer received a score of one, resulting in a maximum score of 13. Total scores of <5, 5–8, and >8 were considered to indicate poor, acceptable, and high levels of knowledge, respectively.

*Ethics*

Ethical approval for this study was obtained from the Research and Ethics Committee of the Directorate General of Planning and Studies, Ministry of Health, Oman (#MOH/CSR/18/9478). All participants were briefed regarding the objectives of the study and were informed that their participation was voluntary and that they had the right to withdraw at any time. Written informed consent was received from all of the subjects prior to their participation in the study. The participants’ anonymity and confidentiality were ensured at all times, with each subject assigned a unique identification number for the purposes of data analysis.

*Statistical analysis*

The data analysis was carried out using the Statistical Package for the Social Sciences (SPSS), version 23 (IBM Corp., Armonk, New York). Descriptive statistics were used to describe the sample characteristics. For categorical variables, frequencies and percentages were reported, whereas means and standard deviations were used to present continuous variables. Pearson’s Chi-squared (*χ*^2^) test or Fisher’s exact test (for low cell frequencies) was used to test significance as appropriate, and a p value of ≤ 0.05 was considered statistically significant. 

## Results

All 805 women selected for inclusion participated in the study (response rate: 100%). The majority were either 21–30 (n = 313; 38.9%) or 31–40 (n = 327; 40.6%) years of age. More than half (n = 420; 52.2%) were educated to the undergraduate level with either a diploma or bachelor’s degree and only 11 (1.4%) had no formal education. A total of 166 participants (20.6%) held healthcare-related degrees. Almost half (n = 368; 45.7%) were employed. Most of the participants were married (n = 627; 77.9%); of these, 450 (71.8%) had gotten married at 18–25 years of age. The total number of children ranged from 1–13, with an average of 2.52 ± 2.44 children and 1.25 ± 1.49 daughters. Approximately one-third of the respondents (n = 270; 33.5%) had an income of 500–1,000 Omani rials; however, most (n = 630; 78.3%) reported that income did not affect their decision to undertake screening. The majority reported that their husbands held either secondary school diplomas (n = 329; 40.9%) or undergraduate degrees (n = 216; 26.8%).

In terms of risk factors for cervical cancer, 257 participants (31.9%) did not engage in regular exercise. Only three (0.4%) were smokers. Most participants (n = 635; 78.9%) did not use birth control methods, although 13 (1.6%) reported taking oral contraceptive pills. Approximately one-third had a history of abortions (n = 242; 30.1%). Nine participants (1.1%) had previously had an STD, of which four (44.4%) had been diagnosed with HPV. A personal and family history of cervical cancer was reported by six (0.7%) and 27 (3.4%) participants, respectively. Five participants (0.6%) reported factors indicative of low immunity, while only one (0.1%) had a husband with low immunity.

Upon assessing cervical cancer-related knowledge, most participants (n = 543; 67.5%) had previously heard of cervical cancer, with the most common source of information being social media (n = 266; 33%), followed by healthcare providers (n = 136; 16.9%), television programs or advertisements (n = 133; 16.5%), schools or universities (n = 104; 12.9%), family and friends (n = 66; 8.2%), and written media such as magazines or newspapers (n = 65; 8.1%). Just over one-quarter (n = 212; 26.3%) believed that cervical cancer was one of the top five leading causes of death worldwide. Spotting between periods (n = 381; 47.3%), vaginal discharge (n = 337; 41.9%), persistent pelvic pain (335; 41.6%), and post-menopausal bleeding (314; 39.0%) were frequently identified warning signs of cervical cancer. In addition, just under half of the participants identified persistent low back pain (n = 381; 47.3%), menorrhagia (n = 313; 38.9%), postcoital bleeding (n = 286; 35.5%), dyspareunia (n = 279; 34.7%), and weight loss (n = 250; 31.1%) to be warning signs. However, blood in the stool and urine (n = 211; 26.2%) and diarrhea (n = 99; 12.3%) were infrequently identified as warning signs of cervical cancer. 

The most frequently identified risk factors of cervical cancer were immunosuppression (n = 458; 56.9%), a family history of cervical cancer (n = 429; 53.3%), and a lack of regular screening (n = 400; 49.7%). Moderately identified risk factors included smoking (n = 387; 48.1%), HPV infection (n = 313; 38.9%), long-term use of combined oral contraceptives (n = 295; 36.6%), and low immunity on the part of the husband (n = 277; 34.4%). Early marriage (n = 137; 17%) and having three or more children (n = 75; 9.3%) were the risk factors least commonly associated with cervical cancer.

With regards to knowledge of Pap smear testing, 410 participants (50.9%) had previously heard of this screening method, with the source of information usually being healthcare services (n = 167; 20.7%), followed by social media (n = 127; 15.8%), television programs or advertisements (n = 46, 5.7%), family and friends (n = 43; 5.3%), school or universities (n = 42; 5.2%), and written media (n = 38; 4.7%). Most participants (n = 514; 63.9%) agreed that Pap smear testing involved the collection of a sample from the cervix, although some believed the sample was collected from the vagina (n = 119; 14.8%). Approximately half of the participants did not know the ideal frequency of Pap smear testing (n = 374; 46.5%), that it was important that such testing be performed regularly (n = 403; 50.1%), or the meaning of an abnormal Pap smear result (n = 370; 46%). 

The majority of participants were unaware of the availability of Pap smear testing in Oman (n = 424; 52.7%); in fact, a portion believed that such testing was reserved only for married women (n = 186; 23.1%) or those who were postmenopausal (n = 373; 46.3%). Most participants (n = 654; 81.2%) admitted that they did not take part in Pap smear testing on a regular basis. Only 126 participants (15.7%) had themselves previously taken part in Pap smear testing, of which 62 (53%) had done so between 21–30 years of age. Various reasons were reported for having undertaken Pap smear testing, including being reminded by their doctors (n = 69; 54.7%), understanding its importance (n = 46; 36.5%), reaching the designated age for screening (n = 37; 29.3%), and having a family history (n = 13; 10.3%) or personal history (n = 7; 5.5%) of cervical cancer. 

Among those who had never undertaken Pap smear testing, less than half of the participants were willing to do so in the future (n = 290; 42.7%) or to do so on a regular basis (n = 283; 41.7%). Reasons for being reluctant to undertake Pap smear testing included not being familiar with it (n = 437; 54.3%), fear of the test itself (n = 155; 19.3%), being unmarried (n = 144; 17.9%), privacy concerns (n = 114; 14.2%), having no time (n = 112; 13.9%), and fear of the results (n = 102; 12.7%). Most participants stated that abnormal Pap smear results should be followed by a biopsy (n = 488; 60.6%), blood test (n = 463; 57.5%), colposcopy (n = 436; 54.2%), another Pap smear test (n = 427; 53%), and HPV testing (n = 398; 49.4%). 

In terms of cervical cancer, 318 participants (39.5%) demonstrated poor knowledge (scores of <7), 379 (47.1%) had acceptable knowledge (scores of 7–14), and 108 (13.4%) had high knowledge (scores of >14). Cervical cancer knowledge scores were significantly associated with education level (p < 0.001), whether their degree was related to healthcare (p < 0.001), income (p < 0.001), and employment status (p < 0.001) (Table 1). Regarding Pap smear testing, 313 participants (38.9%) demonstrated poor knowledge (scores of <5), 404 (50.2%) had acceptable knowledge (scores of 5–8), and 88 (10.9%) had high knowledge (scores of >8). Pap smear knowledge scores were significantly associated with age (p = 0.001), educational qualification (p < 0.001), whether their qualification was related to healthcare (p < 0.001), employment status (p <0.001), marital status (p = 0.003), and income (p < 0.001) (Table 2). 

No significant associations were noted between knowledge scores for either cervical cancer or Pap smear testing and other sociodemographic characteristics, the presence of a family history of cervical cancer, or the presence of a personal history of STDs, HPV infection, or cervical cancer (Tables 1 and 2). Furthermore, no significant associations were noted between Pap smear practice or willingness to undertake Pap smear testing in future and any sociodemographic characteristics, the presence of a family history of cervical cancer, or the presence of a personal history of STDs, HPV infection, or cervical cancer (Tables 3 and 4). 

**Table 1:** Association between Sociodemographic and Clinical Characteristics and Knowledge Regarding Cervical Cancer among the Participants (N = 805)

Characteristic	Knowledge scores, n (%)			*P*-value
	Poor	Acceptable	High	
Age (years) (n = 798)				
≤ 30	143 (38.9)	180 (48.9)	45 (12.2)	0.52
31–40	130 (39.8)	153 (46.8)	44 (13.5)	
> 40	41 (39.8)	43 (41.7)	19 (18.4)	
Education level (n = 802)				
Illiterate	6 (54.5)	4 (36.4)	1 (9.1)	< 0.001
Primary	16 (47.1)	14 (41.2)	4 (11.8)	
Secondary	152 (49)	134 (43.2)	24 (7.7)	
Undergraduate	140 (33.3)	210 (50)	70 (16.7)	
Postgraduate	2 (7.4)	16 (59.3)	9 (33.3)	
Healthcare-related degree (n = 804)				
No	287 (45)	290 (45.5)	61 (9.6)	< 0.001
Yes	31 (18.7)	88 (53)	47 (28.3)	
Employment status (n = 790)				
Student	35 (51.5)	29 (42.6)	4 (5.9)	< 0.001
Unemployed	161 (46.9)	156 (45.5)	26 (7.6)	
Employed	108 (29.4)	182 (49.6)	77 (21)	
Retired	3 (25)	8 (66.7)	1 (8.3)	
Marital status (n = 802)				
Single	63 (46.3)	58 (42.6)	15 (11)	0.678
Married	239 (38.1)	300 (47.8)	88 (14)	
Widowed	5 (38.5)	7 (53.8)	1 (7.7)	
Divorced	11 (42.3)	11 (42.3)	4 (15.4)	
Monthly income (OMR) (n = 632)				
< 500	74 (45.4)	78 (47.9)	11 (6.7)	< 0.001
500–1,000	119 (44.1)	119 (44.1)	32 (11.9)	
1,000–2,500	45 (27.4)	87 (53)	32 (19.5)	
> 2,500	5 (14.3)	17 (48.6)	13 (37.1)	
Personal history of STDs (n = 805)				
No	315 (39.6)	373 (46.9)	108 (13.6)	0.359
Yes	3 (33.3)	6 (66.7)	0 (0)	
Personal history of HPV infection (n = 805)				
No	316 (39.5)	378 (47.2)	107 (13.4)	0.628
Yes	2 (50)	1 (25)	1 (25)	
Personal history of cervical cancer (n = 805)				
No	316 (39.5)	375 (46.9)	108 (13.5)	0.115
Yes	2 (33.3)	4 (66.7)	0 (0)	
Family history of cervical cancer (n = 805)				
No	314 (40.4)	360 (46.3)	104 (13.4)	0.023
Yes	4 (14.8)	19 (70.4)	4 (14.8)	

OMR, Omani riyals; STDs, sexually transmitted diseases; HPV, human papillomavirus.

**Table 2:** Association between Sociodemographic and Clinical Characteristics and Knowledge Regarding Papanicolaou Smear Testing among the Participants (N = 805)

Characteristic	Knowledge scores, n (%)			*P*-value
	Poor	Acceptable	High	
Age (years) (n = 798)				
≤ 30	151 (41)	193 (52.4)	24 (6.5)	0.001
31–40	110 (33.6)	166 (50.8)	51 (15.6)	
> 40	47 (45.6)	43 (41.7)	13 (12.6)	
Education level (n = 802)				
Illiterate	8 (72.7)	3 (27.3)	0 (0)	< 0.001
Primary	17 (50)	15 (44.1)	2 (5.9)	
Secondary	147 (47.4)	145 (46.8)	18 (5.8)	
Undergraduate	134 (31.9)	227 (54)	59 (14)	
Postgraduate	5 (18.5)	13 (48.1)	9 (33.3)	
Healthcare-related degree (n = 804)				
No	288 (45.1)	309 (48.4)	41 (6.4)	< 0.001
Yes	25 (15.1)	94 (56.6)	47 (28.3)	
Employment status (n = 790)				
Student	32 (47.1)	36 (52.9)	0 (0)	< 0.001
Unemployed	172 (50.1)	155 (45.2)	16 (4.7)	
Employed	97 (26.4)	201 (54.8)	69 (18.8)	
Retired	4 (33.3)	6 (50)	2 (16.7)	
Marital status (n = 802)				
Single	69 (50.7)	63 (46.3)	4 (2.9)	0.003
Married	229 (36.5)	319 (50.9)	79 (12.6)	
Widow	7 (53.8)	5 (38.5)	1 (7.7)	
Divorced	6 (23.1)	16 (61.5)	4 (15.4)	
Monthly income (OMR) (n = 632)				
< 500	74 (45.4)	80 (49.1)	9 (5.5)	< 0.001
500–1,000	117 (43.3)	133 (49.3)	20 (7.4)	
1000–2,500	41 (25)	87 (53)	36 (22)	
> 2,500	5 (14.3)	22 (62.9)	8 (22.9)	
Personal history of STDs (n = 805)				
No	309 (38.8)	400 (50.3)	87 (10.9)	0.936
Yes	4 (44.4)	4 (44.4)	1 (11.1)	
Personal history of HPV infection (n = 805)				
No	310 (38.7)	403 (50.3)	88 (11)	0.317
Yes	3 (75)	1 (25)	0 (0)	
Personal history of cervical cancer (n = 805)				
No	310 (38.8)	403 (50.4)	86 (10.8)	0.115
Yes	3 (50)	1 (16.7)	2 (33.3)	
Family history of cervical cancer (n = 805)				
No	304 (39.1)	389 (50)	85 (10.9)	0.826
Yes	9 (33.3)	15 (55.6)	3 (11.1)	

OMR, Omani riyals; STDs, sexually transmitted diseases; HPV, human papillomavirus

**Table 3:** Association between Sociodemographic and Clinical Characteristics and Previous Experience Undergoing Papanicolaou Smear Testing among the Participants (N = 805)

Characteristic	Previously undergone Papanicolaou smear testing		*P*-value
	No	Yes	
Age (years) (n = 798)			
≤ 30	307 (83.4)	61 (16.6)	0.432
31–40	283 (86.5)	44 (13.5)	
> 40	85 (82.5)	18 (17.5)	
Education level (n = 802)			
Illiterate	10 (90.9)	1 (9.1)	0.865
Primary	27 (79.4)	7 (20.6)	
Secondary	259 (83.5)	51 (16.5)	
Undergraduate	357 (85)	63 (15)	
Postgraduate	23 (85.2)	4 (14.8)	
Healthcare-related degree (n = 804)			
No	540 (84.6)	98 (15.4)	0.634
Yes	138 (83.1)	28 (16.9)	
Employment status (n = 790)			
Student	58 (85.3)	10 (14.7)	0.993
Unemployed	288 (84)	55 (16)	
Employed	308 (83.9)	59 (16.1)	
Retired	10 (83.3)	2 (16.7)	
Marital status (n = 802)			
Single	111 (81.6)	25 (18.4)	0.828
Married	532 (84.8)	95 (15.2)	
Widow	11 (84.6)	2 (15.4)	
Divorced	22 (84.6)	4 (15.4)	
Monthly income (OMR) (n = 632)			
< 500	142 (87.1)	21 (12.9)	0.491
500–1,000	228 (84.4)	42 (15.6)	
1,000–2,500	137 (83.5)	27 (16.5)	
> 2,500	27 (77.1)	8 (22.9)	
Personal history of STDs (n = 805)			
No	671 (84.3)	125 (15.7)	0.706
Yes	8 (88.9)	1 (11.1)	
Personal history of HPV infection (n = 805)			
No	676 (84.4)	125 (15.6)	0.606
Yes	3 (75)	1 (25)	
Personal history of cervical cancer (n = 805)			
No	673 (84.2)	126 (15.8)	0.29
Yes	6 (100)	0 (0)	
Family history of cervical cancer (n = 805)			
No	654 (84.1)	124 (15.9)	0.23
Yes	25 (92.6)	2 (7.4)	

OMR, Omani riyals; STDs, sexually transmitted diseases; HPV, human papillomavirus

**Table 4:** Association between Sociodemographic and Clinical Characteristics and Willingness to Undergo Papanicolaou Smear Testing among the Participants (N = 805)

Characteristic	Willingness to undergo Papanicolaou smear testing		*P*-value
	No	Yes	
Age (years) (n = 798)			
18–20	35 (4.4)	20 (2.5)	0.891
21–30	195 (24.4)	118 (14.8)	
31–40	213 (26.7)	114 (14.3)	
> 40	67 (8.4)	36 (4.5)	
Education level (n = 791)			
Illiterate or primary	22 (64.7)	12 (35.3)	0.691
Secondary	205 (66.1)	105 (33.9)	
Undergraduate or postgraduate	282 (63.1)	165 (36.9)	
Healthcare-related degree (n = 804)			
No	409 (64.1)	229 (35.9)	0.952
Yes	106 (63.9)	60 (36.1)	
Employment status (n = 790)			
Student	40 (58.8)	28 (41.2)	0.41
Unemployed	228 (66.5)	115 (33.5)	
Employed	228 (62.1)	139 (37.9)	
Retired	9 (75)	3 (25)	
Marital status (n = 802)			
No	86 (10.7)	50 (6.2)	0.82
Yes	428 (53.4)	238 (29.7)	
Monthly income (OMR) (n = 632)			
< 500	101 (62)	62 (38)	0.865
500–1,000	173 (64.1)	97 (35.9)	
1,000–2,500	104 (63.4)	60 (36.6)	
> 2,500	20 (57.1)	15 (42.9)	
Personal history of STDs (n = 805)			
No	510 (64.1)	286 (35.9)	0.597
Yes	5 (55.6)	4 (44.4)	
Personal history of HPV infection (n = 805)			
No	511 (63.8)	290 (36.2)	0.132
Yes	4 (100)	0 (0)	
Personal history of cervical cancer (n = 805)			
No	510 (63.8)	289 (36.2)	0.321
Yes	5 (83.3)	1 (16.7)	
Family history of cervical cancer (n = 805)			
No	496 (63.8)	282 (36.2)	0.481
Yes	19 (70.4)	8 (29.6)	

OMR, Omani riyals; STDs, sexually transmitted diseases; HPV, human papillomavirus

## Discussion

Although cervical cancer is one of the most common cancers in Omani women, there is as yet no established screening program in the country (Catalan Institute of Oncology/International Agency for Research on Cancer Information Centre on HPV and Cancer, 2019). In Oman, Pap smear testing is usually offered only to married women; moreover, it is primarily performed for diagnostic purposes at secondary and tertiary care institutes, and remains unavailable entirely at the primary healthcare level. In our study, the majority of participants (67.5%) had heard of cervical cancer, while around half (50.9%) had heard of Pap smear testing. This level of general awareness is low compared to a previous study performed in Oman which found that the majority of participants (80%) had heard of cervical cancer (Nasar et al., 2016). This difference in findings could be due to the fact that the previous study was conducted among patients, students, and employees at a tertiary care institute that routinely provides this service.

Shrestha et al., (2013) found that 65.7% of women visiting a tertiary care center in Nepal had heard of cervical cancer, although only 18.1% were aware of Pap smear testing. In contrast, much higher rates were reported among women visiting primary healthcare centers in Qatar (85.2% and 76.4%, respectively) (Al-Meer et al., 2011). In Kuwait, 76.9% of married women attending polyclinics had heard of Pap smear testing (Al Sairafi and Mohamed, 2009). In the USA, reported results from Vietnamese American women revealed that 90% had heard of cervical cancer, while 74% had heard of the Pap test (Nguyen et al., 2002). Variations in these results may be due to differences in study population, design, and the availability of cervical cancer screening in various countries. In the current study, knowledge of cervical cancer and Pap smear testing was significantly influenced by certain sociodemographic variables, including education level, employment status, and income. In particular, participants who held college degrees and were employed tended to demonstrate higher levels of knowledge compared to their counterparts. Similar findings have been reported in studies from Qatar, Kuwait, and the USA (Nguyen et al., 2002; Al Sairafi and Mohamed, 2009; Al-Meer et al., 2011).

For the participants in our study, the main source of information regarding cervical cancer was social media (33%), with only 16.9% and 12.9% receiving information on this topic from healthcare providers and school and universities, respectively. Likewise, the most common sources of information regarding Pap smear testing were healthcare providers (20.7%) and social media (15.8%). Given the varied sources of information reported, as well as the discrepancies in knowledge of cervical cancer warning signs and risk factors, these findings could highlight that—even if Omani women attending primary healthcare centers have heard of cervical cancer and Pap screening—they might not have received accurate information concerning these subjects. As such, the development of a national well-structured awareness program is highly recommended. Such an initiative should be provided by healthcare professionals and could utilize social media channels in order to disseminate important cervical cancer-related information to the public. In addition, collaboration with the Ministry of Education could aid in the integration of appropriate health education into the national curricula, including warning signs and risk factors of cervical cancer and the importance of regular screening to ensure early diagnosis and treatment.

Only 15.7% of the Omani women attending primary healthcare centers in our cohort revealed that they had previously undergone Pap smear testing. Among those who had, the majority were aged 31–40 years, educated to the undergraduate level, and married. In contrast, uptake rates among those with a personal history of STDs, HPV infection, and cervical cancer and those with a family history of cervical cancer were 11%, 25%, 0%, and 7.4%, respectively. Although these risk factors should theoretically increase Pap smear uptake, it is possible that these participants were lost to follow-up or were not instructed appropriately by their physicians regarding the importance of regular Pap smear testing. 

Moreover, among the women in the present study who had never undertaken Pap smear testing, only 36% were willing to undergo such testing in the future. Commonly reported barriers to taking part in such screening included being unfamiliar with the test, fear of the procedure itself, and privacy concerns. It is likely that these women would demonstrate more positive attitudes concerning Pap smear testing if they were to be given more detailed information concerning the test. Therefore, it is imperative that the health sector take action to educate and reassure this group concerning Pap smear testing procedures. Much of this responsibility is likely to fall on attending physician who should provide detailed information to female patients regarding cervical cancer screening, including its indications, ideal frequency, and possible complications. Moreover, all healthcare professionals-whether at the primary, secondary, or tertiary care level-should seek to maintain appropriate channels of communication and education with their patients throughout the entire consultation and follow-up process.

This study had various strengths and limitations. To the best of the authors’ knowledge, it is the first study conducted in Oman to assess knowledge, attitudes, and practices regarding cervical cancer screening among Omani women attending primary healthcare centers. As such, the findings provide important baseline information which may help to design and select an appropriate public health and educational initiatives to deliver important information concerning cervical cancer and Pap smear testing to members of the general public. Moreover, the choice of face-to-face interviews as the primary method of data collection may have helped to build a stronger rapport with the respondents. 

However, as the study targeted solely women attending primary healthcare centers, the generalizability of the results for those in the general community was limited. In addition, such individuals may have had medical-related issues which might contribute to selection bias. However, all participants had undergone triage and were waiting in the waiting area, indicating that they were not critically ill and were clinically stable; in addition, they all confirmed that they were well enough to participate in the face-to-face interviews. Finally, the inclusion of questions referring to the participants’ past experiences and the face-to-face interview setup itself could have inadvertently led to recall or response bias.

In conclusion, knowledge regarding cervical cancer and Pap smear testing was suboptimal among a cohort of Omani women attending multiple local primary care centers across the country. Hence, the Ministry of Health is strongly urged to consider initiating a nationwide screening program in order to reduce the incidence of cervical cancer. In addition, there is an urgent need for well-structured national awareness and educational programs. Such initiatives could be delivered using a multimedia approach involving social media channels in order to deliver appropriate information regarding the importance of cervical cancer screening to the community at large. In addition, the involvement of other governmental sectors, such as the Ministry of Education, would be invaluable to help target younger women as well as to include information regarding cervical cancer warning signs and risks factors within the national curricula. Further studies are recommended to help inform the implementation of such educational initiatives.

## Author Contribution Statement

T.K., M.R., and R.K. conceived the presented research idea and went through literature review. T.K. and M.R under the supervision of R.K. designed the research methodology and the questionnaire format. T.K. and M.R were involved in the data collection and date entry. T.K., M.R., and R.K analyzed and interpreted the results. T.K. was a major contributor in writing the manuscript in consultation with M.R. and R.K. R.K was the research supervisor who guided T.K. and M.R. throughout the project. All authors read and approved the final manuscript.

## Data Availability

The datasets used and/or analyzed during this study are available from the corresponding author upon reasonable request.

## List of abbreviations

AIDS: acquired immunodeficiency syndrome; CIN: cervical intraepithelial neoplasia; HIV: human immunodeficiency virus; HPV: human papillomavirus; Pap: Papanicolaou; STDs: sexually transmitted diseases; USA: United States of America.
